# Stimulus-dependent spatial patterns of response in SI cortex

**DOI:** 10.1186/1471-2202-6-47

**Published:** 2005-07-19

**Authors:** Joannellyn S Chiu, Mark Tommerdahl, Barry L Whitsel, Oleg V Favorov

**Affiliations:** 1 Departments of Biomedical Engineering, University of North Carolina at Chapel Hill, Chapel Hill, North Carolina 27599, USA; 2 Cell and Molecular Physiology, University of North Carolina at Chapel Hill, Chapel Hill, North Carolina 27599, USA

## Abstract

**Background:**

Recently we reported that vibrotactile flutter stimulation of a skin locus at different amplitudes evokes an optical response confined to the same local region of the primary somatosensory cortex (SI), where its overall magnitude varies proportionally to the flutter amplitude. In this report, we characterize the impact of the flutter amplitude on the spatial patterns of activity evoked within the responding SI region.

**Results:**

In order to characterize the spatial pattern of activity within the responding SI region, images of the flutter-evoked SI optical response were segmented and analyzed with spatial frequency analysis. The analysis revealed that: (1) dominant spatial frequencies in the optical intrinsic signal emerge within the responding SI region within 3–5 sec of stimulus onset; (2) the stimulus-evoked activity is spatially organized in a form of several roughly parallel, anterior-posteriorly extended waves, spaced 0.4–0.5 mm apart; (3) the waves themselves exhibit spatial periodicities along their long axis; and (4) depending on the flutter stimulus amplitude, these periodicities can range from fine 0.15 mm "ripples" at 50 μm amplitude to well-developed 0.5 mm fluctuations at the amplitude of 400 μm.

**Conclusion:**

The observed spatiointensive fractionation on a sub-macrocolumnar scale of the SI response to skin stimulation might be the product of local competitive interactions within the stimulus-activated SI region and may be a feature that could yield novel insights into the functional interactions that take place in SI cortex.

## Background

Afferent projections from skin to primary somatosensory cortex (SI) are well known to form a fine map of the body surface in SI. In this map, a skin locus provides afferent input to an extensive cortical region in SI [1,2]. In particular, the direct connectivity between somatosensory thalamus and SI cortex is now recognized to be much more spatially distributed than previously believed (e.g., in primates the ventrobasal thalamic region which receives its input from a single digit projects to an extensive, 20 mm^2 ^sector of SI cortex – [3,4]). The intrinsic SI excitatory connections link not only neighboring but also widely separated regions of somatosensory cortex [5]. These connections ensure that many members of widely distributed neuronal populations interact extensively within milliseconds after the onset of stimulus-evoked thalamocortical drive. Thus it is not surprising to find that the processing of even a very local skin stimulus is associated with SI activation over several sq. millimeters of cortical area, as revealed, for example, with optical imaging techniques [6-10].

Such spatially extensive cortical regions are not functionally homogeneous. For example, using Optical Intrinsic Signal (OIS) imaging in near-infrared (830 nm) range, we find that in squirrel monkeys a small-diameter stimulus probe oscillating on the skin at 25 Hz activates more than 3 mm^2 ^of cortical territory in area 3b of SI [6,8,11]. Such a territory can contain as many as 20 place-defined cortical columns ("segregates"; [12]) organized into 4–6 alternating rapidly- and slowly-adapting submodality bands [13]. Chen et al. [10] reported that the relative magnitudes of optical response in local, 0.2–0.4 mm wide, SI regions changes when the frequency of the stimulating probe is changed from simple taps to 25 Hz to 200 Hz (thus preferentially activating different submodalities of skin mechanoreceptors). And on even finer spatial scale SI might be organized in ~50 μm-diameter functionally distinct minicolumns [1,12,14-18].

Together these considerations suggest that the spatial pattern of activity evoked in SI by even the smallest stimuli might be structurally more complex than a typically envisioned basic bell-shaped pattern. A closer inspection of such patterns might reveal certain spatial formations within them with significant functional implications. Recently, we investigated the response of SI cortex to varying amplitudes of flutter stimulation. Regardless of the amplitude of stimulation (in the range of 50 to 400 μm), we found that the spatial extent of the response of SI cortex remained the same [19]. Instead, the actuated cortical region exhibits increases in its magnitude of neuronal response proportional to the intensity of stimulation [19-21]. One feature of particular interest in our study was that the activity patterns evoked within these spatially delineated regions, when viewed at high resolution, appeared to develop in an orderly manner dependent on stimulus amplitude. The purpose of this study was to determine if those patterns are indeed systematic, and if so, to characterize them quantitatively.

## Results

**Figure 1:**
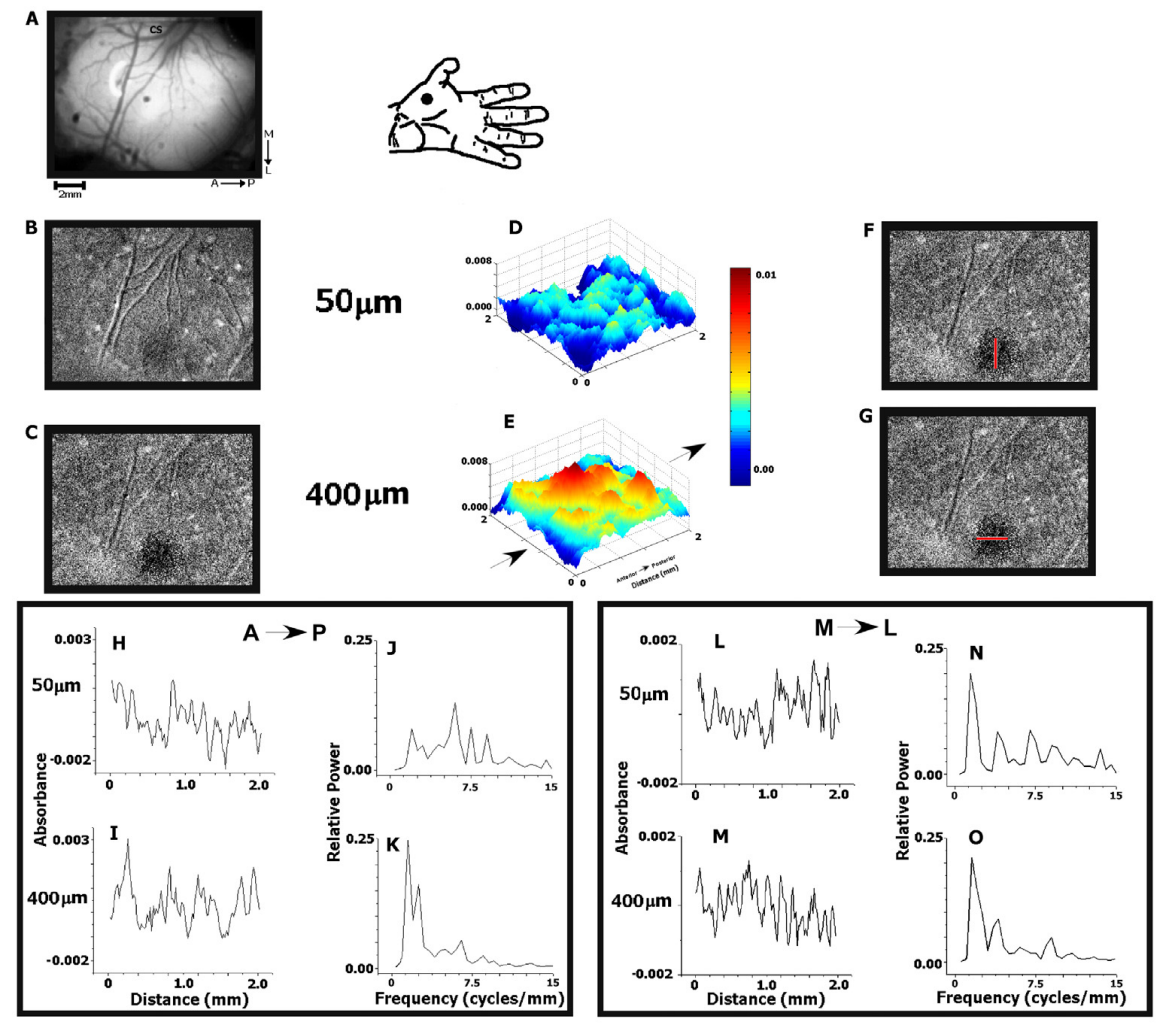
Comparison of SI cortical responses evoked by low- and high-amplitude 25 Hz flutter stimuli. **A: **View of the somatosensory cortex with the lateral end of the central sulcus (CS) at the top. Stimulus location is shown on the hand figurine. **B: **Optical response of the SI to the low-amplitude (50 μm peak-to-peak) flutter. Shown is the light absorbance image ([poststimulus image – prestimulus image]/prestimulus image) averaged across 20 stimulus trials, and in each trial across 3 poststimulus images taken during the 5–7 sec interval of continuous stimulation. Increased light absorbance is indicated by darker shading. **C: **Optical response of SI to the high-amplitude (400 μm) flutter. **D & E: **Magnified 3-D surface maps of OIS magnitude in the responding cortical region. **F & G**: Orientation of segmentation in the M-L dimension (F) and the A-P dimension (G). **H, I, L & M**: Spatiointensive histograms generated via segmentation of areas indicated in F & G for two different stimulus conditions. **J, K, N & O: **Periodograms showing the spatial frequency composition of the spatiointensive histograms. Note change in the power spectra with the change in stimulus amplitude in data sampled in the A-P orientation, but not in the M-L orientation.

Figure 1 illustrates the basic method that we used to examine and compare the absorbance patterns evoked by different amplitudes of flutter stimulation. Panel A shows the cortical field (SI cortex of squirrel monkey) that was imaged. Panels B and C are the light absorbance images evoked in this subject by low-amplitude (50 μm) and high-amplitude (400 μm) flutter stimulation of the same spot on the thenar eminence. The responses to both stimuli occupy the same approximately circular, 2 mm-diameter cortical region. The stimulus-evoked activity in the central 2 × 2 mm sector of this responding region was plotted as a 3-dimensional surface map (Panels D and E) to facilitate the view of the evoked patterns of response. In order to quantify this pattern, the pattern was segmented along the cortical anterior-posterior axis and the medial-lateral axis (segmentation orientations indicated by Panels F and G) and generated spatial histograms (Panels H, I, L and M). These histograms hint at a spatial pattern that could have some underlying spatial frequency. To observe their spatial frequency composition, the power spectra of these spatial histograms are plotted in Panels J, K, N and O. The periodograms in these panels reveal major differences in the spatial organization of the SI responses to the 50 μm and 400 μm stimuli when segmented in the anterior-posterior dimension. That is, the SI response to the higher-amplitude stimulus is greatly dominated by lower spatial frequencies between 1.5–2.5 cycles/mm, whereas in the SI response to the weaker stimulus the relative power in these frequencies is greatly reduced and greater power is present at spatial frequencies between 5.5–9 cycles/mm. In contrast, when segmented in the medial-lateral dimension (Panels N and O), there is no shift in the power spectra between the low and high amplitude responses. The medial-lateral power spectra are clearly dominated by the low spatial frequencies around 1.5–2.5 cycles/mm.

**Figure 2:**
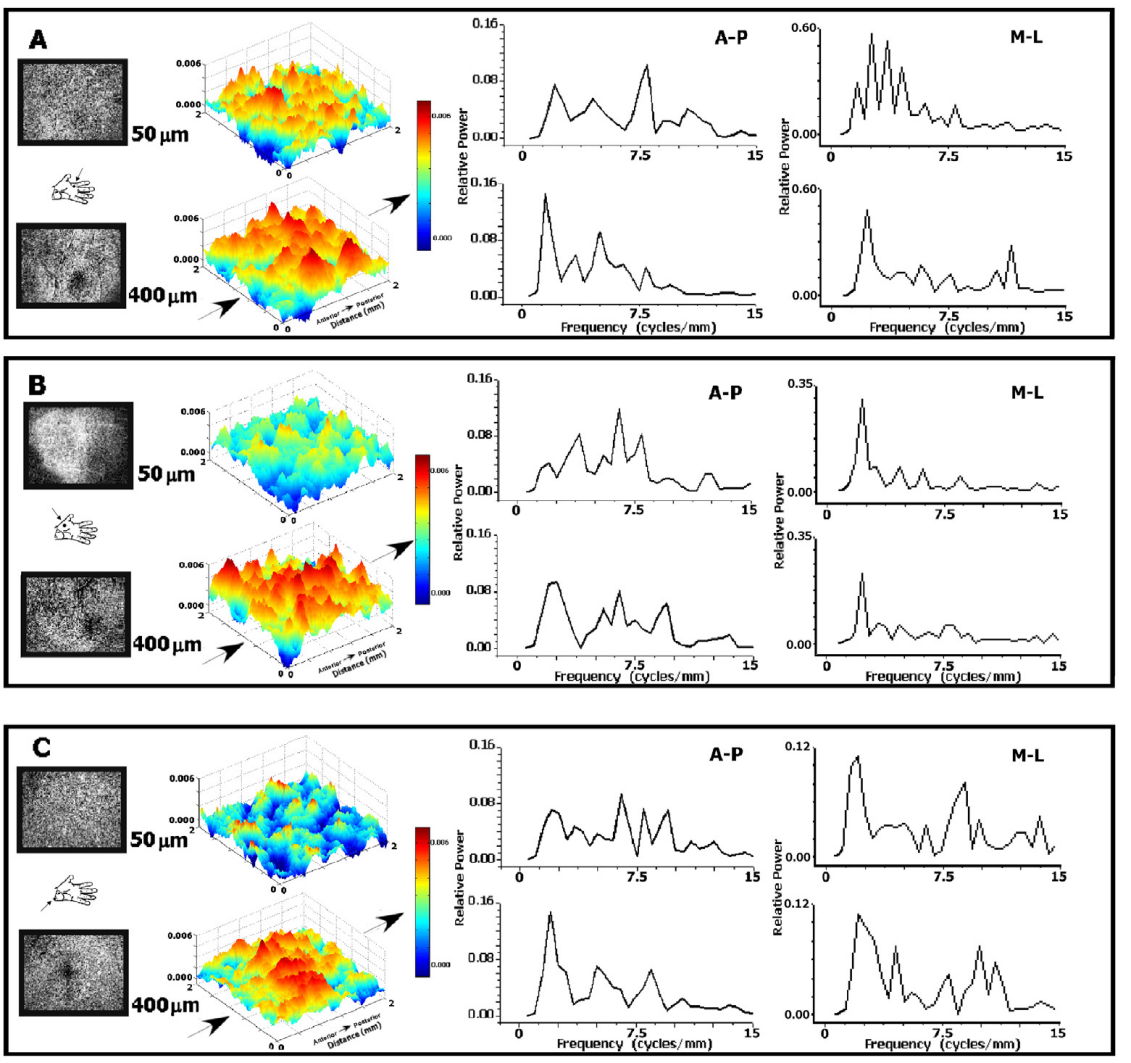
Comparison of SI cortical responses evoked by low- and high-amplitude 25 Hz flutter stimuli in three additional subjects. Shown for each subject (A-C) are: (1) the stimulus location on the hand, (2) the average light absorbance images of the cortical optical response to the 50 μm- and 400 μm-amplitude stimuli, (3) the magnified 3-D activity maps of the region sampled, and (4) the power spectra of the OIS responses. All subjects exhibited similar differences between their low and high stimulus amplitude power spectra.

Comparable results were obtained from the 4 other subjects, (3 subjects shown Fig. 2). Viewing the 3-D activity maps (Figure 2, second-left column), it is evident that the low amplitude stimulus evokes a pattern with a much higher spatial frequency than that evoked by the higher amplitude stimulus. The third column also shows that in each subject there was a shift of the most prominent frequency band of the OIS power spectrum from ~7.5 cycles/mm to ~2 cycles/mm as the stimulus amplitude was increased from 50 μm to 400 μm, when the stimulus-evoked activity was sampled in the anterior-posterior dimension. In the medial-lateral dimension (Figure 2, right column), no such shift in the power spectra was observed. Low frequencies around 2–2.5 cycles/mm dominate all of these spectra.

**Figure 3:**
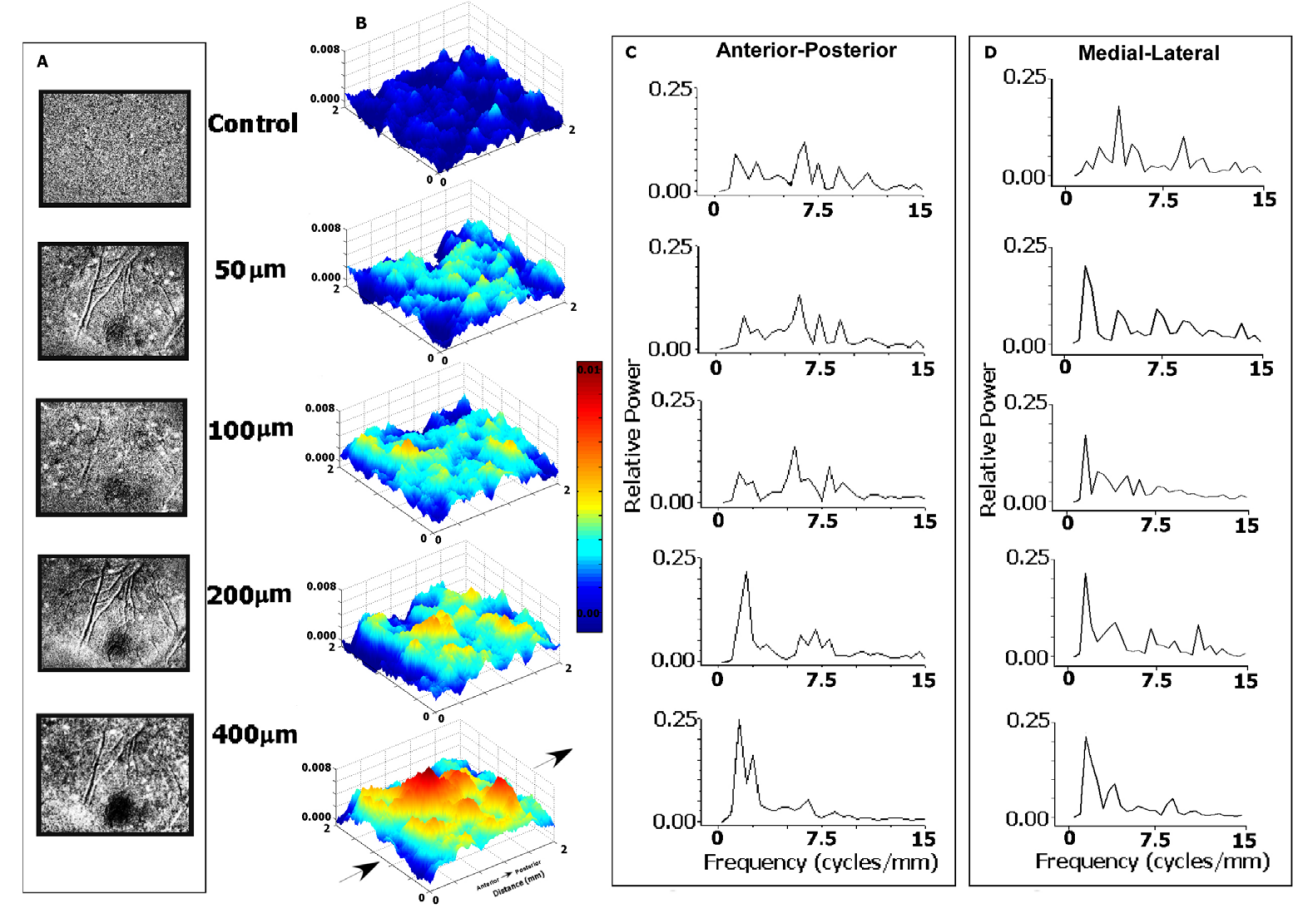
Comparison of SI optical responses evoked at different stimulus amplitudes (0, 50, 100, 200, and 400 μm) in an exemplary experiment. **A: **Average light absorbance images for each of the stimulus amplitudes (20 trials, 3 poststimulus images per trial). **B**: 3-D activity maps of the responding cortical region. **C & D: **Power spectra measured for each stimulus amplitude in the two dimensions sampled. With an increase in stimulus amplitude, the spectral power shifts to lower spatial frequencies for the data sampled in the A-P dimension, but not in the M-L dimension.

Figure 3 illustrates results obtained in a more detailed examination of the effect of increased amplitude of flutter stimulation on the spatial organization of the OIS response pattern. Figure 3A shows the SI optical responses, obtained in the same experiment, to five different stimulus amplitudes ranging from 0 (control) to 400 μm. 3-D surface maps of the central 2 × 2 mm activated cortical region are displayed in Figure 3B. The power spectra of the responses sampled in the anterior-posterior dimension (Fig. 3C) show that in the absence of flutter (0 μm stimulus amplitude) the distribution of power at different spatial frequencies is relatively uniform, with the most dominant frequency at 6.5 cycles/mm. With increments in stimulus amplitude, however, the relative power in the spectral distribution shifts towards lower spatial frequencies. In the case of the medial-lateral dimension (Figure 3D), the power spectra remain essentially constant, with an exception of the no-stimulus control, and there does not appear to be a shift in the distribution of the power with increasing the amplitude of stimulation.

**Figure 4:**
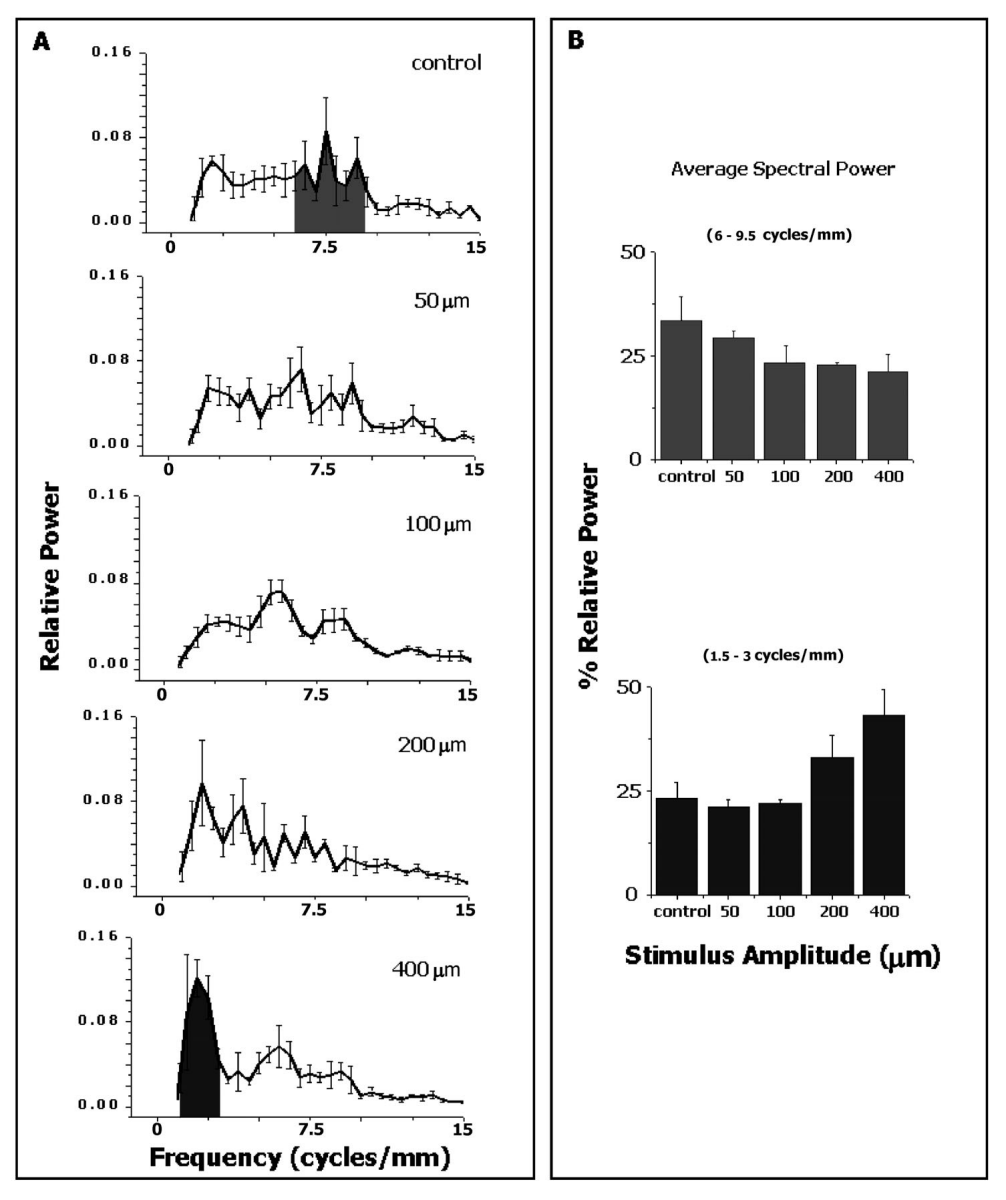
Across-subject reproducibility of the effect of flutter stimulus amplitude on the power spectra of the data sampled in the A-P dimension. **A: **Average periodograms of the OIS responses of 5 subjects to 5 different stimulus amplitudes. **B: **Average relative power in the 6–9.5 cycles/mm and 1.5–3 cycles/mm frequency bands plotted as a function of the stimulus amplitude. As the stimulus amplitude increases, the spectral power shifts progressively to lower spatial frequencies.

**Figure 5:**
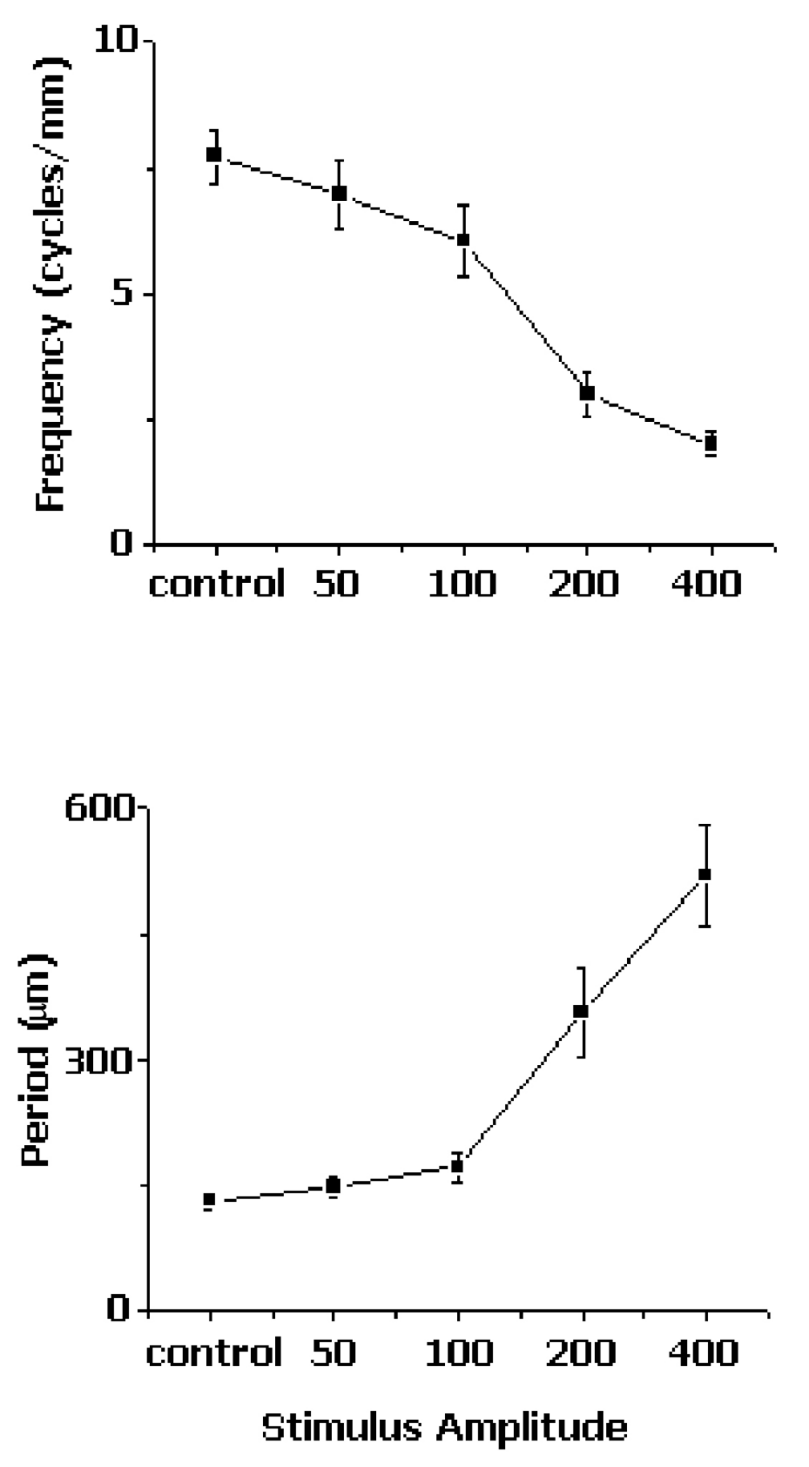
Dependence of the position of the largest spectral peak in OIS periodograms on the stimulus amplitude. The frequency (**A**) and the period (**B**) of the largest spectral peak (average of 5 subjects – see Figure 4) are plotted as a function of the stimulus amplitude. The peak frequency/period shifts across the power spectrum with a change of stimulus amplitude.

This effect of stimulus amplitude on the distribution of spatial frequencies in the OIS response along the anterior-posterior dimension is highly reproducible across all subjects. Figure 4A shows the average across-subject (n = 5) power spectra, obtained in response to flutter stimuli delivered at 5 different amplitudes. The 6–9.5 cycles/mm frequency band, which is most prominent in the absence of stimulation (control; 0 μm amplitude), loses relative power as stimulus amplitude is increased (Fig. 4B top). In contrast, relative power at 1.5–3 cycles/mm grows as stimulus amplitude is increased, and at the highest amplitude used (400 μm) it greatly dominates the power spectrum (Fig. 4B bottom). Overall, the OIS power spectrum appears to respond to an increase in stimulus amplitude by a shift of the relative power towards lower frequencies. This tendency is expressed more clearly in Figure 5, where the highest-power frequency (Fig. 5A; or, inversely, in Fig. 5B, the highest-power period) is plotted as a function of the stimulus amplitude.

**Figure 6:**
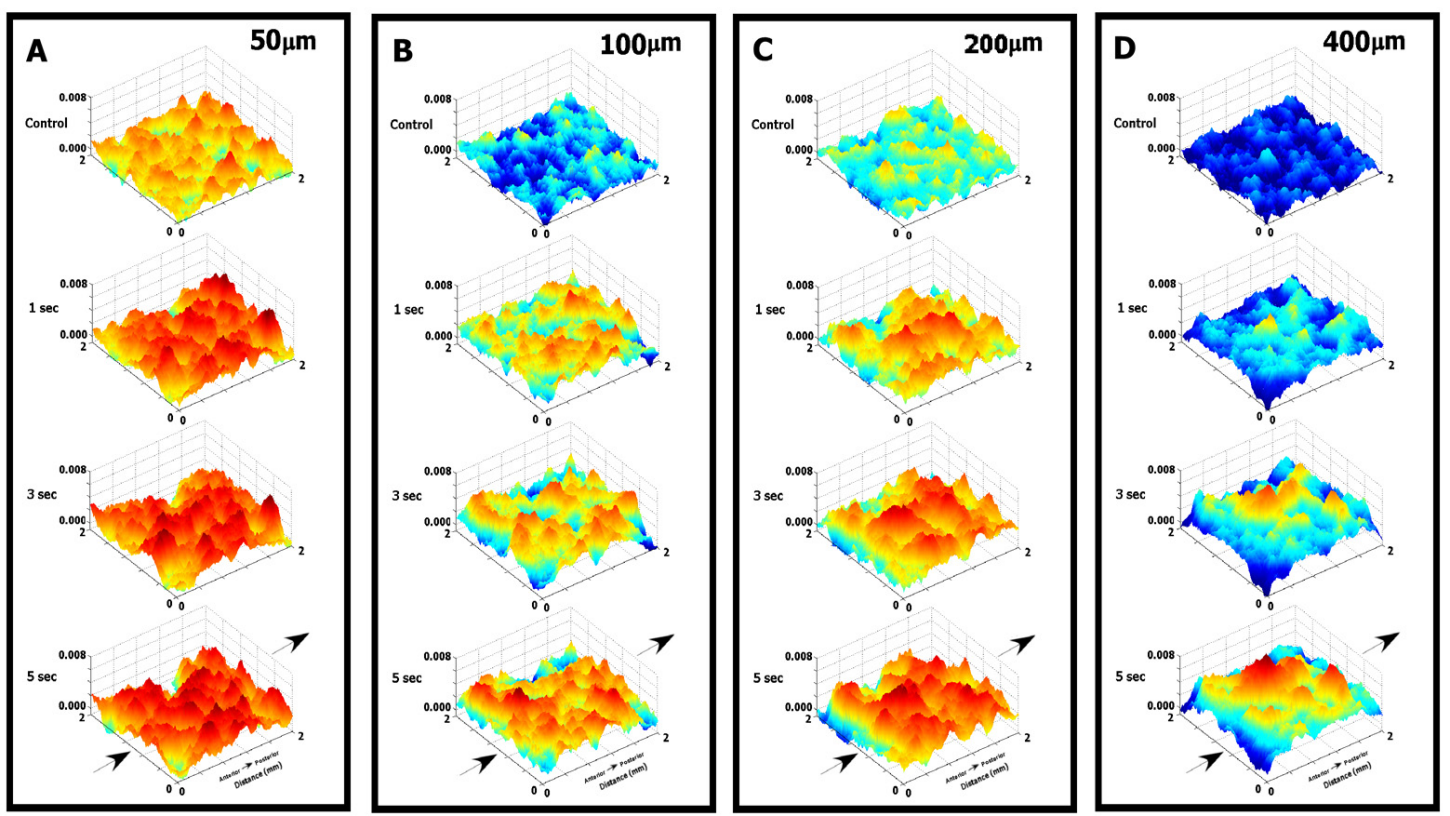
Comparison of temporal development of cortical patterns of absorbance evoked by different amplitudes of flutter vibration. 3-D activity maps of the same cortical region (of the same subject) are plotted at -1 (control), 1, 3 and 5 secs after the stimulus onset for amplitudes of 50, 100, 200 and 400 μm (Panels A, B, C, & D respectively; in all panels, color-scales are normalized by their maxima). Note the difference in the progression of the pattern development evoked by the different stimulus amplitudes.

**Figure 7:**
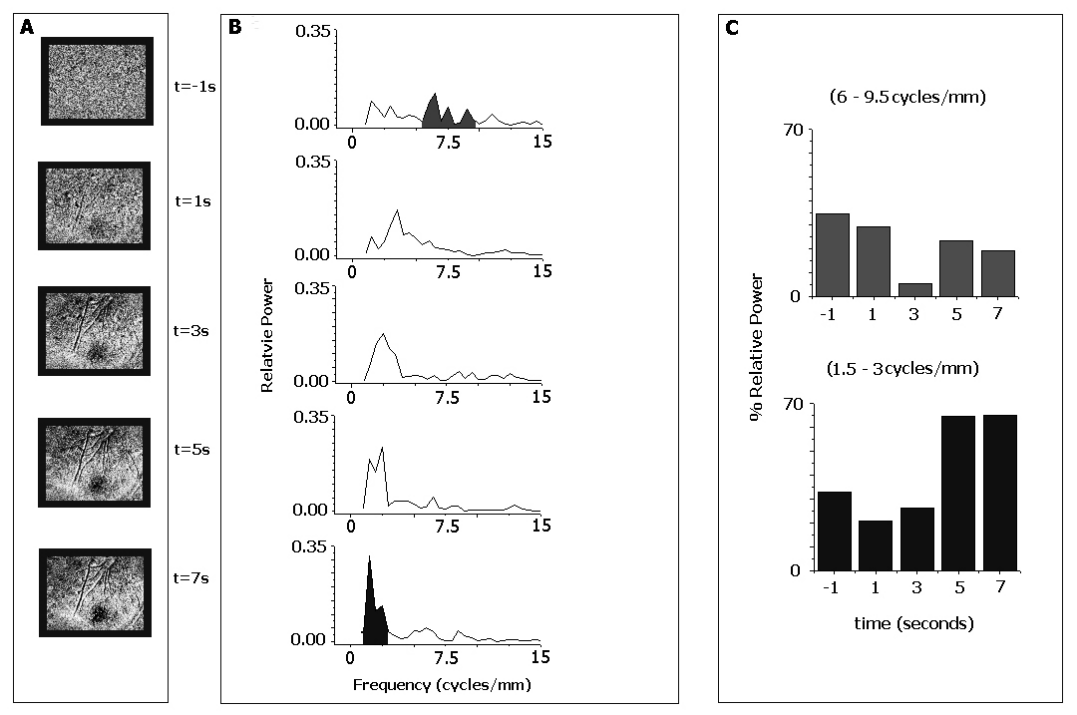
Temporal development of power spectra in response to a 400 μm-amplitude flutter stimulus. **A**: Light absorbance images obtained at selected times before and during flutter stimulation. **B**: Power spectra of OIS activity sampled in the anterior-posterior dimension across the activated cortical region. With increasing stimulus duration, the most prominent frequency band in the power spectra shifts to the left relative to the pre-stimulus condition. **C: **Temporal shift in relative power in the 6–9.5 cycles/mm band (top) and in the 1.5–3 cycles/mm band (bottom). As stimulus duration increases, the relative power decreases in the 6–9.5 cycles/mm band, but increases in the 1.5–3 cycles/mm band.

Stimulus *duration *appears to alter the spatial organization of SI optical response to flutter in a manner similar to the alteration that accompanies an increase in stimulus amplitude. Figure 6 displays the temporal evolution of the responses evoked by four different amplitudes. In each case, the pattern of absorbance evoked by the flutter stimulus appears to become more organized and periodic with time after the stimulus onset. In other words, with increasing stimulus duration, the local aggregates of above background absorbance tend to form larger clusters – which would lead to higher periodic values (lower spatial frequencies). The spatial frequency changes with stimulus duration were quantified in a manner similar to those that were used to quantify the spatial frequency characteristics that changed with stimulus amplitude. To give a representative example, Figure 7 shows the temporal evolution of the SI response of a subject to a 400 μm-amplitude flutter stimulus. The images in Figure 7A were obtained 1 sec prior to stimulus onset (control), as well as at 1, 3, 5, and 7 sec after the onset of continuous skin flutter stimulation. The power spectra obtained from these images (Fig. 7B) show a systematic leftward shift of the dominant frequencies with increasing time after stimulus onset, from ~6.5 cycles/mm prior to stimulation, to ~2 cycles/mm after 7 sec of continuous stimulation. The plots in Figure 7C show that the 6–9.5 cycles/mm frequency band, which is dominant in the resting state, loses relative power after onset of stimulation, and during this same time relative power within 1.5–3 cycles/mm frequency band also becomes maximal.

**Figure 8:**
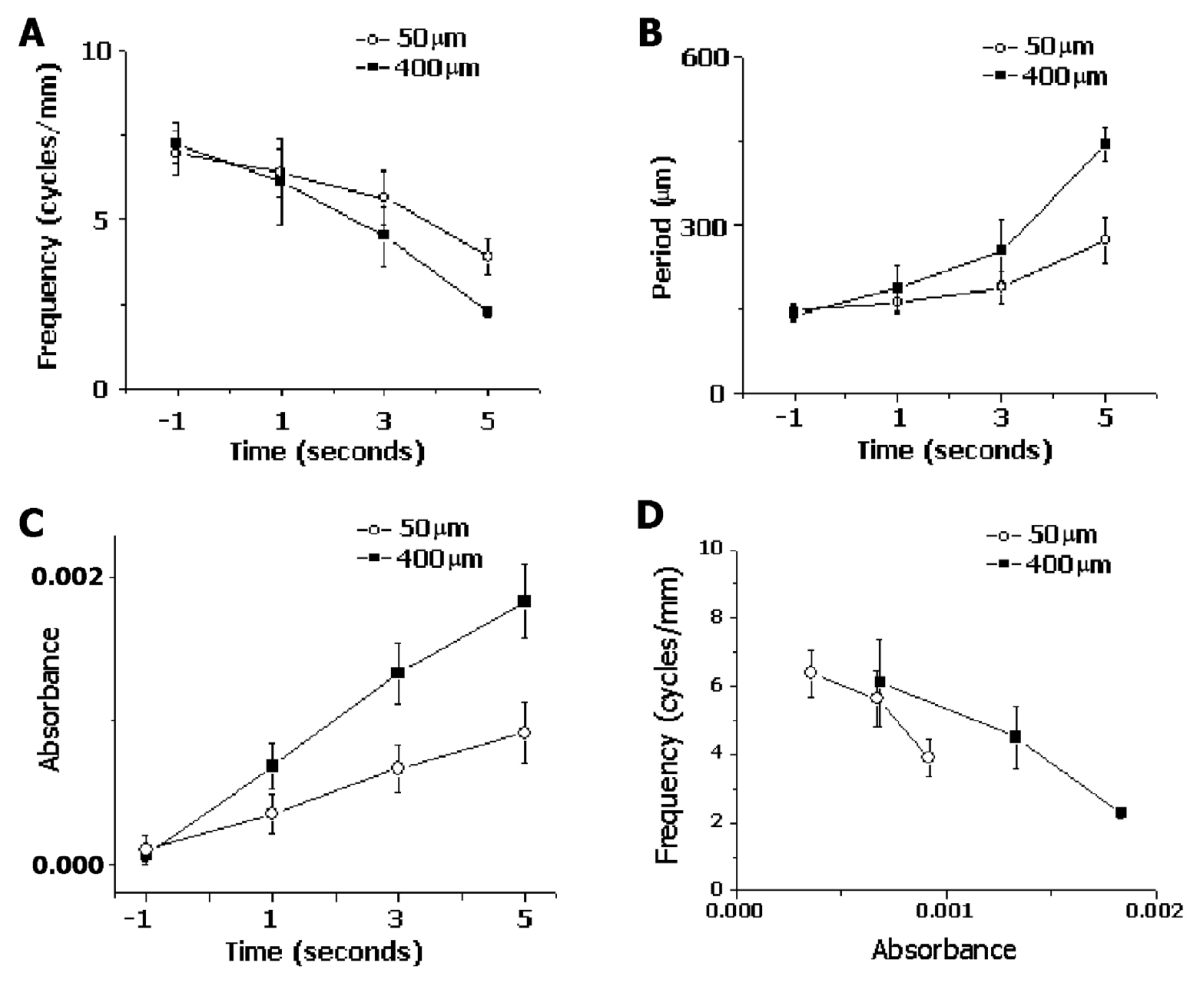
Temporal shift of the position of the largest spectral peak in the anterior-posterior OIS periodograms and its dependence on stimulus amplitude. The frequency (**A**) and the period (**B**) of the largest spectral peak (average of 5 subjects) are plotted as a function of time since the stimulus onset. Two plots are shown, for 50 μm-amplitude and 400 μm-amplitude stimuli. With increasing stimulus duration, the peak frequency/period shifts across the power spectrum, at a rate dependent on the stimulus amplitude. **C: **Average magnitude of OIS in the segmented region as a function of time after stimulus onset. The dominance of lower spatial frequencies develops in time in parallel with an overall growth of the OIS response to the stimulus. **D: **The frequency of the largest spectral peak plotted as a function of the average magnitude of OIS in the sequential region. The relationship between the largest spectral peak and overall OIS magnitude varies depending on the stimulus amplitude.

Figure 8 summarizes the temporal development of OIS response patterns by plotting the highest-power spatial frequency in the anterior-posterior dimension (averaged across 5 subjects) at different times after stimulus onset. Two plots are shown for comparison: the first for 400 μm-amplitude stimuli, the second for 50 μm-amplitude stimuli. These plots make it evident that during continuous flutter stimulation: (1) the spectral power of the response migrates towards lower frequencies with increasing time after stimulus onset – indicating that SI optical response pattern undergoes a gradual spatial reorganization; and (2) the higher the stimulus amplitude, the faster the shift of spectral power towards lower frequencies.

Figure 8C plots the magnitude of OIS response to the 50 μm- and 400 μm-amplitude stimuli as a function of time after the stimulus onset, showing that during the time when the dominant spatial frequency migrates across the power spectrum, the OIS also grows overall in its magnitude. The concurrency of these changes raises a parsimonious possibility that OIS periodicity is a direct function of the OIS magnitude (and thereby only an indirect function – via their control of the OIS magnitude – of the stimulus strength and duration). To evaluate this possibility, Figure 8D plots the highest-power spatial frequency at any given time (taken from Figure 8A plot) as a function of the overall OIS magnitude at that time (taken from the Figure 8C plot). As the Figure 8D plot shows, the relationship between OIS magnitude and periodicity obtained with the 50 μm-amplitude stimuli appears to be different from the relationship obtained with the 400 μm stimuli, suggesting that OIS periodicity cannot be explained simply by the overall OIS magnitude.

## Discussion

Spatial frequency analysis revealed that the SI response to flutter stimulation produces systematic, spatially periodic fluctuations in the magnitude of the OIS within the responding SI region. In the absence of stimulation – the control condition – the power spectra have a small prevalence of spatial periodicities in the range of 0.1–0.14 mm. In contrast, in the presence of a prominent flutter stimulus spatial periodicities in the range of 0.4–0.6 mm dominate the stimulus-activated SI region.

Visual inspection of the 3-D activity plots (Figures 1, 2, 3, 6), together with the results of spectral analyses performed along the anterior-posterior and medial-lateral cortical dimensions, point to a substantial tendency of the stimulus-evoked activity to take a form of a pattern of roughly parallel elongated waves oriented in the anterior-posterior direction. These waves have a medio-lateral spacing of 0.4–0.5 mm between their crests. This tendency is already well developed in response to a relatively weak, 50 μm-amplitude flutter. At such weaker stimuli, each anterior-posteriorly oriented wave has a pattern of fine "ripples" along its long axis, with the dominant spatial periodicities in the neighborhood of 0.15-0.2 mm – values close to those characteristic of the OIS in the no-stimulus condition. Stronger stimuli lead to an emergence of larger spatial periodicities in the anterior-posterior dimension, up to 0.6 mm. Such a stimulus amplitude-dependent shift towards larger spatial periodicities suggests that the spatial pattern of SI response to flutter stimulation undergoes substantial reorganization in response to changes in stimulus amplitude/intensity. That is, the stronger (200–400 μm amplitude) stimuli have an effect of restructuring the anterior-posteriorly oriented waves by replacing the high-frequency ripples along their crests with more prominent *ca. *0.5 mm periodic fluctuations.

Such a reorganization of stimulus-evoked OIS spatial patterns apparently also occurs across time. That is, flutter stimulus-evoked OIS patterns in the anterior-posterior dimension develop gradually from the resting state – dominated by high spatial frequencies – through a series of states characterized by progressively lower spatial frequencies. Thus, the spatial organization of the patterned response evoked in SI by a flutter stimulus varies with both stimulus strength and stimulus duration.

In interpreting the outcomes of our spatial frequency analyses, it is important that it be recognized that they were performed not on the SI neuroelectrical responses to flutter stimulation, but on SI *optical *responses to such stimulation. It now is well established that cortical neuroelectrical activity is positively and strongly correlated with the local increase in cortical tissue light absorbance [8,11,22-27]. Although highly correlated with neuroelectrical activity, however, the OIS is not a direct reflection of either neuronal spike discharge activity or, more generally, the local voltage changes evoked by a sensory stimulus. Notably, it has a much slower onset and decay than the neuroelectrical responses of cortical cells. The intrinsic signal detected using the near-infrared light ("IR imaging") is relatively independent of changes in blood flow [28,29]. The optical signal observed under near-infrared light reflects a variety of factors but, most significantly, change (shrinkage) in the volume of the extracellular fluid compartment attributable to the glial swelling due to stimulus-evoked changes in extracellular [K+] and/or neurotransmitter release [23,30,31]. In view of these complex origins of the near-infrared OIS, it remains to be determined whether the observed stimulus-dependent periodicities reflect the spatial organization of stimulus-evoked neuroelectrical activity. Alternatively, the observed OIS periodicities might reflect the spatial organization of the cortical glial reaction to local neuroelectrical activity, or SI microvascular responses [32], which become more prominent with increases in both stimulus strength and duration.

If the observed stimulus-dependent OIS periodicities were to reflect neuroelectrical activity patterns, how well would they fit with the known features of SI functional organization? In particular, a macrocolumnar functional organization has been well documented in SI cortex. Receptive field-mapping techniques have revealed that SI cortex is partitioned into ~0.5 mm-wide submodality- and place-defined columns [1,2,12,13,33,34]. These topographic entities, repeating every 0.5 mm, might indeed be responsible for the prominent 0.5 mm periodicity in SI OIS stimulus-evoked activity patterns. If the spatial OIS periodicities were due to selective modulation of entire macrocolumns (in other words, due to SI response fractionation on the macrocolumnar scale), then the observed OIS periodicities would be at least double the size of macrocolumns. Since this is not the case, and the OIS periodicities approximate the size of macrocolumns, the fractionation of the SI response would appear to take place on the submacrocolumnar scale, with flutter stimuli preferentially activating only a subsector of each macrocolumn in the SI region engaged by the stimulus. Such preferentially activated subsectors would then form a roughly periodic pattern across the responding SI region. In support of this proposal, Chen et al. [10] have reported that single skin taps, 25 Hz flutter, and 200 Hz vibration (stimulus conditions that allow preferential activation of the different classes of skin mechanoreceptors) were associated with preferential activation of different 0.25 mm-diameter regions within the responding SI territory. The small size of these regions suggests that they should occupy only a subsector of a typical submodality column, rather than a whole column [13].

Whether or not the 0.5 mm periodicities in OIS stimulus-response patterns in SI owe their existence to submodality- and/or place-defined macrocolumns, the fact remains that a flutter stimulus activates multiple cortical loci, separated from each other by less active loci. Such a locally selective distribution of activity in SI is suggestive of the presence in SI functional architecture of a mapping factor in addition to submodality and place on the skin – a factor specific to some yet to be explored attribute(s) of mechanical skin stimulation. Similarly, Bruno and colleagues recently demonstrated that individual barrels in rat SI contain minicolumns of neurons preferring the same whisker deflection angle and that these angular tuning domains could be the result of convergent inputs from thalamocortical cells with corresponding angular preferences [35]. It is possible that upon further investigation, the spatial activity patterns evoked in SI cortex, such as the amplitude-dependent patterns described in this report, will also be found to be submodality-dependent as well.

Finally, considering that spatial periodicities in SI OIS response patterns emerge gradually, and evolve with time after the stimulus onset, the fine sculpting of the SI response might be the result of a network-level neurocomputational process that involves competitive and cooperative interactions among local neuronal aggregates. That is, SI sensitivity to the stimulus attribute(s) responsible for the observed fractionation of activity within the responding SI region might be an emergent property of the SI network (i.e., a product of network-level computation), rather than a simple outcome of selective convergence of thalamocortical afferents on SI neurons.

## Conclusion

Observations of the spatial patterns of SI cortical response within an activated region, such as those evoked by flutter stimulation of the skin, suggest that evoked cortical activity within such a territory is not evenly distributed. Furthermore, the cortical activity patterns change in a manner that appears to be dependent upon stimulus conditions. The observed spatiointensive fractionation on a sub-macrocolumnar scale of the SI response to skin stimulation might be the product of local competitive interactions within the stimulus-activated SI region, and as such can lead to new insights about the functional interactions that take place in the SI cortex.

## Methods

All methods and procedures are consistent with USPHS policies and guidelines on animal care and welfare in biomedical research, and were reviewed and approved in advance by an institutional animal use committee (IACUC). Experiments were conducted in 5 squirrel monkeys. Surgical procedures were carried out under deep general anesthesia (1–4% halothane in a 50/50 mixture of oxygen and nitrous oxide). After induction of anesthesia the trachea was intubated to facilitate positive pressure ventilation and delivery of the gaseous general anesthetic. A catheter was inserted into a branch of the femoral vein of the hindlimb ipsilateral to the hemisphere to be imaged, allowing intravenous (IV) administration of drugs and fluids (5% dextrose and 0.9% NaCl). Methylprednisolone sodium succinate (20 mg/kg) and gentamicin sulfate (2.5 mg/kg) were injected intramuscularly to lessen the probability of halothane-induced cerebral edema and prevent bacterial septicemia, respectively.

A 1.5 cm diameter opening in the skull exposed the forelimb region of SI cortex. A recording chamber was positioned over the opening and cemented to the skull with dental acrylic. The chamber was filled with artificial cerebrospinal fluid, the dura mater overlying SI cortex incised and removed, and all wound margins outside the chamber dressed with long-lasting local anesthetic in oil (Cetacaine). All skin and muscle incisions were closed with sutures and bandaged.

After the completion of all surgical procedures subjects were immobilized with Norcuron (vercuronium bromide; 0.5 mg/kg loading dose; 0. 25-0.5 mg/kg/hr maintenance dose) and ventilated with a gas mixture (a 50/50 mix of oxygen and nitrous oxide; supplemented with 0.5–1.0% halothane). At these concentrations and under normocapnic conditions, halothane has no effect on brain energy metabolism [36,37], and only minor effects on cerebrovascular regulation [38]. Ventilator rate and volume were adjusted to maintain end-tidal CO2 between 3.0–4.0%. EEG and cardiovascular signs (EEG slow wave content; EKG and heart rate) were monitored continuously, and the anesthetic gas mixture adjusted intermittently to maintain values and reactivity to skin stimuli consistent with light general anesthesia. Rectal temperature was maintained (using a heating pad) at 37.5°C.

The recording chamber was filled with artificial cerebrospinal fluid and hydraulically sealed using a clear glass plate. Vibrotactile stimuli were delivered to selected loci on the hand using a servocontrolled vibrotactile stimulator [39], capable of delivering precisely controlled sinusoidal vertical skin displacement stimuli. The stimulator made contact with the skin via a cylindrical 2 mm-diameter Delrin probe. All sinusoidal vibrotactile stimuli were superimposed on a static displacement ("pedestal") of 500 μm. Identical parameters of stimulation were used at each skin site that was studied: frequency of vibration 25 Hz (in the flutter range), stimulus duration 7 sec, and interstimulus interval 60 sec. Different peak-to-peak amplitudes of flutter stimulation (0, 50, 100, 200 and 400 μm) were interleaved on a trial-by-trial basis.

The optical imaging system consisted of a computer-interfaced CCD camera, light source, guide and filters required for near-infrared (830 nm) illumination of the cortical surface, a focusing device, and a recording chamber capped by an optical window (for additional methodological details see [7,40]). Images of the exposed cortical surface were acquired 200 ms before stimulus onset ("reference" or "prestimulus" images) and continuously thereafter ("poststimulus" images; at a resolution of one image/s) for 15s following stimulus onset. Exposure time was 200 ms. Light absorbance images were generated by subtracting each prestimulus (reference) image from a poststimulus image and subsequently dividing by the reference image. Absorbance images obtained in this way typically show regions in which light absorption increases and other regions in which absorption decreases in response to skin stimulation. These regions, respectively, have been shown to correspond to regions in which neuronal activity increases and decreases in response to sensory stimulation ([6,27,41-43]; for review see [44]). Stimulus-evoked OIS responses of SI were displayed as either grayscale images or 3-D surface plots. To reduce amount of noise in these displays, the images were smoothed using a 3 × 3 pixel boxcar filter.

Cortical images were taken at light/time exposures that place the region of interest in the middle of the range of the recorded pixel values. Histogram analysis was used during experimental setup to make sure to avoid any nonlinearities that may arise from overexposure. In some of the experiments the camera was rotated by 90° relative to the SI orientation to better capture the responding cortical field. These rotations did not have any noticeable effect on spectral power distributions along the anterior-posterior and medial-lateral cortical dimensions.

The spatial organization of the stimulus-related light absorbance changes in SI was evaluated using linear image segmentation. This involved segmentation of the relevant region of the image into a linear series of bins and computation of the average absorbance value of the pixels in each bin. The sequence of average absorbance values obtained in this way was plotted as a function of distance (mm) along the cortical path traced by the central points of the series of bins – yielding an absorbance *vs. *distance plot (thus forming a spatial histogram). Power spectra of the spatial histograms were then computed using Discrete Fourier Transform (DFT) algorithm and plotted as a periodogram. Fourier analysis was always performed on raw, unfiltered images.

At the end of the experiment the subject was euthanized by overdose of pentobarbital (50 mg/kg/IV), followed by intracardial perfusion with saline and 10% formalin.

## Authors' contributions

JC participated in acquisition of optical data, performed analysis of the data and drafted the manuscript. OF and BW participated in the design of the study, the conduct of the experiments and the drafting of the manuscript. MT participated in the design, conduct, and analysis of the experiments, and in the manuscript preparation.
